# Anxiety and depression in thymoma patients in China before surgery

**DOI:** 10.1186/s13019-022-02081-5

**Published:** 2022-12-16

**Authors:** Jiaduo Li, Guoyan Qi, Yaling Liu

**Affiliations:** Center of Treatment of Myasthenia Gravis, People’s Hospital of Shijiazhuang Affiliated to Hebei Medical University, Shijiazhuang, Hebei Province China

**Keywords:** Thymoma, Anxiety, Depression, Related factors

## Abstract

**Background:**

The study's goal was to investigate the percentage of anxiety and depression in Chinese thymoma patients before surgery, and also the factors that influence it.

**Methods:**

The study included patients who had an anterior mediastinal mass discovered by chest CT and were scheduled for video-assisted thoracoscopic surgery. The mental health rating scales were completed by all patients before surgery. Patients were divided into two groups based on the Hospital Anxiety and Depression Scale (HADS): anxiety/depression and non-anxiety/depression. The association between thymoma clinical factors and the HADS score was studied statistically.

**Results:**

The study comprised eighty patients with thymoma. Before the operation, 22.5% (18/80) of the patients had anxiety and/or depression. The resigned coping style characteristics, along with myasthenia gravis (MG), were associated with preoperative anxiety and depression. The greater the score of the resigned dimension, the greater the risk of anxiety and depression, based on the results of logical regression analysis. Thymoma patients with myasthenia gravis have a higher risk of anxiety and depression.

**Conclusion:**

Patients with myasthenia gravis and resigned coping style were found to have higher anxiety and depression before surgery for Chinese thymoma patients.

## Introduction

Patients with malignant tumors most often suffer from anxiety and depression, which can compromise their tolerance to the tumor and affect the outcome of treatment [1]. In the past several years, an increasing number of studies have started paying attention to the consequences of anxiety and depression in patients with tumors. In order to understand the role of anxiety and depression in malignant tumors, it is necessary to first determine their percentage and the variables that affect them. The studies on the incidence and contributing variables of anxiety and depression in malignant tumors have been increasing, including lung cancer, gastric cancer, breast cancer, and so on [2–4]. However, in thymoma, there is a lack of related research. Thymoma is the most common anterior mediastinal tumor, and nearly 30% of thymoma patients are complicated with myasthenia gravis [5–7]. Patients with myasthenia gravis have an increased risk of depression, and its severity is significantly correlated with depression, according to previous research [8, 9]. However, many of these studies were associated with thymoma, and the results were limited to the relationship between myasthenia gravis and depression, ignoring the effect of thymoma on psychological factors. Therefore, it is necessary to carry out relevant investigations to understand the psychological factors of patients with thymoma. The study's primary aim is to evaluate the preoperative anxiety and depression of thymoma patients and explore the related influencing factors.

## Methods

### Patients and methods patients

Patients with anterior mediastinal tumors who received treatment at the Thoracic Surgery Department, People’s Hospital of Shijiazhuang, Hebei Province, from October 2020 to October 2021 were comprised in this cross-sectional research. The following were the criteria for inclusion: (I) anterior mediastinal mass was discovered by chest CT before surgery, and thymoma was diagnosed clinically and pathologically after surgery; (II) age > 18; (III) thoracoscopy surgery was planned; (IV) agreed to participate and cooperate with the inquiry. The following were the exclusion criteria: (I) inability to communicate accompanied by psychological or mental illness; (II) using antianxiety or antidepressants within one week of the study’s start; (III) history of a malignant tumor. The Ethics Committee of People’s Hospital of Shijiazhuang has given this study its ethical approval (Approval number 202094). All of the patients gave their informed consent orally and were approved by the ethics committee. All methods were performed in accordance with the Declaration of Helsinki.

### Investigation

On the day before surgery, patients in this study completed an independent self-questionnaire. If the patient has difficulty filling out the questionnaire, the medical staff will assist them in completing it. Their sociodemographic status (e.g., age, gender, insurance status), personal and family history, and lifestyle were all noted on the questionnaire. The following are the questionnaires that were utilized in our research.

The hospital Anxiety and Depression Scale (HADS) is divided into two subscales that assess anxiety and depression [10]. In our research, the Chinese version of HADS was used. HADS is a reliable and valid measure of anxiety and depression [11, 12]. Each subscale has 7 items with scores varying between 0 to 21. A score of 11–21 indicates anxiety or depression. Higher anxiety or depression levels are indicated by higher scores. Patients were separated into two groups according to their HADS scores. The anxiety/depression group included people who had anxiety and/or depression, while the non-anxiety/depression group included those who did not have anxiety or depressive symptoms.

The Medical Coping Mode Questionnaire (MCMQ) is a tool for determining a patient’s coping style [13]. In Chinese cancer patients, the MCMQ has been frequently employed [14, 15]. The MCMQ consists of 20 items with scores ranging from 1 (never) to 4 (very high), split into three dimensions: confrontive, avoidant, and resigned. The higher a patient’s score on a certain dimension, the more likely he or she is to pick a coping mode.

The Social Support Rating Scale (SSRS) is a tool for determining social assistance. The validity and reliability of the SSRS scale are excellent [16]. The survey consists of ten questions. The questions ask about people's social support at three levels: objective support, subjective support, and support utilization. Higher scores indicate that the individual receives more social support outside of the immediate family.

The type D scale-14 (DS-14) was used to evaluate type D personality [17]. Two subscales of the DS-14 were used to assess this: negative affectivity (NA) and social inhibition (SI). Each subscale has seven items. Previous studies have shown that DS-14 has high reliability and validity when it was translated into Chinese [18].

### Statistical analyses

All the analysis on the data were conducted using SPSS 21.0 for Windows. For descriptive statistics, the mean, standard deviation (SD), number (n), and percentage were utilized. Continuous data were compared using the non-paired t-test. Comparing categorized data was done with the χ2 test and Fisher's exact test. The clinical factors were studied using logistic regression analysis. Any clinical factors with a P < 0.05 in the univariate analysis were included in the multivariate logistic regression analysis. A significance level of 0.05 was used to determine if the results were statistically significant.

## Results

Eighty-four patients diagnosed with anterior mediastinal mass preoperatively participated in our research. According to the postoperative pathology, 80 cases were diagnosed as thymoma, 3 cases were thymic cysts, and 1 case was teratoma. These 80 patients with thymoma became the final study subjects. Table 1 showed their demographic and medical characteristics. The presence of preoperative anxiety and depression before surgery in thymoma patients was 22.5% (18/80). The grouping was determined according to the HADS score. Eighteen patients were classified as being in the anxiety/depression group. Among them, 3 were anxious, 6 were depressed, and 9 were anxious and depressed. The non-anxiety/depression group was made up of the other 62 patients.

**Table 1:** Patients with thymoma's demographic and clinical characteristics, and scale test results

Variables	Non-anxiety/depression group	Anxiety/depression group	*p*
Age (median [IQR])(year)	45.77 [38.12, 52.81]	44.97 [39.19, 50.09]	0.836
Sex = Female/Male (%)	20/42 (32.3/67.7)	8/10 (44.4/55.6)	0.501
Insurence = yes (%)	43 (69.4)	16 (88.9)	0.176
MG = yes (%)	14 (22.6)	9 (50.0)	0.049
*WHO subtypes (%)*			0.916
A	8 (12.9)	2 (11.1)	
AB	2 ( 3.2)	1 ( 5.6)	
B1	15 (24.2)	5 (27.8)	
B2	27 (43.5)	6 (33.3)	
B3	10 (16.1)	4 (22.2)	
*Masaoka stage (%)*			0.972
I	24 (38.7)	6 (33.3)	
II	21 (33.9)	7 (38.9)	
III	13 (21.0)	4 (22.2)	
IV	4 ( 6.5)	1 ( 5.6)	
*Coping mode*			
Confrontive (mean (SD))	17.32 (2.99)	18.67 (3.18)	0.102
Avoidant (mean (SD))	14.58 (2.98)	14.94 (2.15)	0.632
Resigned (median [IQR])	10.00 [9.00, 11.75]	12.00 [11.00, 12.00]	0.001
*Type of support*			
Objective (median [IQR])	10.00 [10.00, 11.00]	10.50 [10.00, 11.00]	0.591
Subjective (median [IQR])	25.00 [22.00, 26.00]	24.50 [23.00, 26.75]	0.651
Utilization (median [IQR])	7.00 [6.00, 8.00]	7.00 [6.00, 8.00]	0.402
TypeD = no/yes (%)	38/24 (61.3/38.7)	6/12 (33.3/66.7)	0.067

*WHO* World Health Organization

There was a significant difference in the proportion of myasthenia gravis between the anxiety and depression group and the non-anxiety and depression group (P < 0.05), as shown in Table 1. The two groups' MCMQs revealed no significant difference between their confrontive and avoidant aspects. On the other hand, the anxiety/depression group's resigned score was much greater than the control group's.

When comparing the SSRS, the findings suggest that they received similar support in terms of objective support, subjective support, and utilization of support. The proportion of Type D personalities was not significantly different between the two groups, according to the DS-14 test findings.

Univariate analysis showed that myasthenia gravis, coping style and type D personality were closely associated with anxiety and depression (P < 0.05), as shown in Table 2. As demonstrated in Table 3, myasthenia gravis and coping style were independent risk variables for anxiety and depression in multivariate analysis.

**Table 2:** Univariate analysis of anxiety/depression among 80 thymoma patients

Variables	POR	95% CI	P value
Age (year)	0.99	0.94–1.05	0.82
Sex	0.6	0.2–1.74	0.34
Medical insurance	3.53	0.74–16.92	0.11
Myasthenia gravis	3.43	1.14–10.29	0.03
*WHO pathological type*			
A	–		
AB	2	0.11–34.82	0.63
B1	1.33	0.21–8.49	0.76
B2	0.89	0.15–5.29	0.9
B3	1.6	0.23–11.08	0.63
*Masaoka stage*			
I	–		
II	1.33	0.39–4.6	0.65
III	1.23	0.29–5.16	0.78
IV	1	0.09–10.66	1
*MOMC*			
Confrontive	1.17	0.97–1.41	0.11
Avoidant	1.05	0.87–1.27	0.63
Rresigned	1.44	1.11–1.88	0.01
*SSRS*			
Objective	1.15	0.73–1.8	0.55
Subjective	1.05	0.85–1.29	0.66
Utilization	0.85	0.61–1.19	0.35
Type D Persionality	3.17	1.05–9.56	0.04

*POR* The prevalence odds ratio

**Table 3:** Multivariate analysis of anxiety/depression among 80 thymoma patients

Variables	POR	95% CI	*P*-value
*Myasthenia gravis*			
No	–		
Yes	5.34	1.49–19.18	0.01
*MOMC*			
Rresigned	1.59	1.17–2.18	0.003

*POR* The prevalence odds ratio

## Discussion

As mentioned in the literature review, stress and depression can lead to impaired immune response and may promote the occurrence and development of tumors [16]. Very little was found in the literature on the question of thymoma and psychological factors. This study is the first to cross-sectionally investigate the percentage of anxiety/depression and its associated variables in thymoma patients.

In China, cancer patients often suffer from both depression and anxiety [1]. In this research, 50% (9/18) of thymoma patients had anxiety-depression comorbidities. Anxiety-depression comorbidities were found to be 45.5 percent in cervical cancer and 68.5 percent in bladder and kidney cancer in previous investigations [19, 20]. This is significant because comorbid mental disorders are connected with more serious symptoms, poorer outcomes, and more medical resource utilization than single mental diseases [21].

In this study, preoperative anxiety and depression were shown to be prevalent in 22.5 percent of thymoma patients, which was lower than that of other different types of tumors [18–20, 22]. The disparity in percentage could be due to two factors. First, thymoma was relatively rare, the incidence rate was 0.15 per 100,000 person-years, so it was easy to be ignored [6]. In addition, a good prognosis may reduce the incidence of anxiety and depression in thymoma. Thymoma has a favorable prognosis, as the five-year overall survival rate was around 90% [21]. Thymoma has a five-year median survival rate of 69 percent in advanced illnesses [23].

Coping style is the key factor affecting the outcome of stressful events. Different coping styles will lead to different emotional states [24]. The anxiety/depression group had a higher resigned dimension than the non-anxiety/depression group in this research. The MCMQ's confrontive and avoidant components, however, showed no significant differences. Patients with diseases with little hope of recovery are more likely to use “resigned” coping strategies [25, 26]. Based on the data, the group in anxiety/depression chose to use the strategy of "resigned". Using this coping strategy may worsen an individual's symptoms of anxiety and depression. Preoperative psychological counseling for patients suffering anxiety and depression may be helpful in assisting them establish a more positive coping style.

Studies have found that social support is a protective factor for mental health [27, 28]. Individuals might benefit from social support to avoid stress and poor health [29, 30]. In various studies, social support has been shown to have a significant role in improving mental health, and this link applies to cancer patients as well [27, 28, 31, 32]. The social support in the two groups was not significantly different in this study. The results of this survey indicate that the needs of thymoma patients in Chinese medical settings are mostly satisfied by three kinds of assistance, namely material, psychological, and therapeutic assistance.

Anxiety and depression are linked to Type D personalities [33]. According to previous research, cancer patients who have type D personalities tended to experience anxiety and depression [34]. In the univariate analysis, the researchers found that the type D personality was associated with anxiety and depression. However, in multivariate analysis, anxiety and depression were not significantly influenced by type D personality. It's thought that the cause of the problem is a combination of type D personality and other factors. Personality types have been proven in some research to predict and influence coping styles [35, 36]. Adaptive personality qualities are significantly related to active coping methods in some studies [37, 38]. The “resigned” coping style is a negative coping style. Maladaptive personality traits and poor coping styles have a high association [39, 40]. Individuals with maladaptive personalities were more likely to experience psychological discomfort because they were likely to utilize a maladaptive coping style, according to the relationship between personality and coping styles.

According to our findings, myasthenia gravis is an independent factor of anxiety and depression. Myasthenia gravis is a chronic disease that hurts one’s life quality and can lead to anxiety and depression. Patients suffering from MG have long been thought to experience depressive symptoms [41, 42]. Previous research has found that the severity of myasthenia gravis is strongly linked to depression [9]. The early improvement of the condition with suitable MG treatment may be helpful for thymoma patients with myasthenia gravis to lessen anxiety and sadness.

There are a few limitations in our research. First, because the sample we studied came from an oncology institution, we should proceed with caution in drawing conclusions from the current findings. Second, the HADS used in this study is mainly used to screen anxiety and depression among patients in general hospitals. Third, more research is needed to see if the findings of the current study apply to other thymoma samples and diverse cultural contexts. Fourth, in addition to coping style, social support, and type D personality, we should further explore the factors related to psychological disorders. Finally, this study employed a cross-sectional approach. As a result, the data cannot be used to infer causality. To confirm the current findings, more longitudinal investigations are needed.

## Conclusion

In summary, this research found that the frequency of preoperative anxiety and depression in Chinese patients with thymoma is low when compared to other tumors, and that its incidence is influenced by coping strategies and myasthenia gravis. The observations indicate that before thymoma surgery, medical staff should provide adequate cognitive coaching so that patients can develop a positive coping style and reduce myasthenia gravis symptoms early.

## Data Availability

The original contributions presented in the study are included in the article, further inquiries can be directed to the corresponding author.
